# Health Surveillance of Wild Brown Trout (*Salmo trutta fario*) in the Czech Republic Revealed a Coexistence of Proliferative Kidney Disease and *Piscine Orthoreovirus-3* Infection

**DOI:** 10.3390/pathogens9080604

**Published:** 2020-07-24

**Authors:** Ľubomír Pojezdal, Mikolaj Adamek, Eva Syrová, Dieter Steinhagen, Hana Minářová, Ivana Papežíková, Veronika Seidlová, Stanislava Reschová, Miroslava Palíková

**Affiliations:** 1 Department of Virology, Veterinary Research Institute, 621 00 Brno, Czech Republic; syrova@vri.cz (E.S.); reschova@vri.cz (S.R.); 2 Fish Disease Research Unit, Institute for Parasitology, University of Veterinary Medicine, 30559 Hannover, Germany; mikolaj.adamek@tiho-hannover.de (M.A.); Dieter.Steinhagen@tiho-hannover.de (D.S.); 3 Department of Ecology and Diseases of Zooanimals, Game, Fish and Bees, Veterinary and Pharmaceutical University, 612 42 Brno, Czech Republic; minarova@vri.cz (H.M.); papezikovai@vfu.cz (I.P.); seidlovav@vfu.cz (V.S.); palikovam@vfu.cz (M.P.); 4 Department of Immunology, Veterinary Research Institute, 621 00 Brno, Czech Republic; 5 Department of Zoology, Fisheries, Hydrobiology and Apiculture, Mendel University, 613 00 Brno, Czech Republic

**Keywords:** immunohistochemistry, infectious hematopoietic necrosis, infectious pancreatic necrosis, PCR, *salmonid alphavirus*, serology, *Tetracapsuloides bryosalmonae*, viral hemorrhagic septicemia

## Abstract

The population of brown trout (*Salmo trutta fario*) in continental Europe is on the decline, with infectious diseases confirmed as one of the causative factors. However, no data on the epizootiological situation of wild fish in the Czech Republic are currently available. In this study, brown trout (n = 260) from eight rivers were examined for the presence of viral and parasitical pathogens. *Salmonid alphavirus-2*, infectious pancreatic necrosis virus, *piscine novirhabdovirus* (VHSV) and *salmonid novirhabdovirus* (IHNV) were not detected using PCR. Cell culturing showed no viruses as well, and serological analysis of 110 sera did not detect any specific antibodies against VHSV or IHNV. Fish from two rivers were positive for the presence of *piscine orthoreovirus-3* (PRV-3), subtype PRV-3b. However, none of the PRV-3-positive fish showed gross pathologies typically associated with PRV infections. By far the most widespread pathogen was *Tetracapsuloides bryosalmonae* which was confirmed in each of the examined locations, with a prevalence of up to 65% and 100%, as established by immunohistochemistry and PCR, respectively. Furthermore, up to 43.8% of fish showed signs of proliferative kidney disease caused by *T. bryosalmonae*, suggesting that this parasite is a main health challenge for brown trout in the Czech Republic.

## 1. Introduction

The number of recirculation and flow-through systems rearing portion-sized salmonids, mostly rainbow trout (*Oncorhynchus mykiss*) and brook trout (*Salvelinus fontinalis*), in the Czech Republic is slowly but steadily increasing, with 1.1 tons of fish produced in 2018 [1]. Infectious diseases, especially those listed by OIE [2], pose an economic threat to aquaculture either directly, via clinical signs, lower weight gain and higher mortality [3], or indirectly, via the costs of surveillance, prevention and eradication measures, which are mandated by European and national legislation. Considering the OIE-listed diseases of viral origin, the Czech Republic is declared free of infectious salmon anemia (ISA), but has a non-defined status considering viral hemorrhagic septicemia (VHS) and infectious hematopoietic necrosis (IHN), with outbreaks of both diseases occurring sporadically throughout the years [4]. The unusually high number of outbreaks of VHS and IHN in the winter of 2013/2014 [5], followed by costly eradication measures, raised the question of the role of wild fish as possible pathogen vectors in the environment of Czech rivers. 

Several pathogens are considered a threat to wild salmonids in continental Europe. *Piscine novirhabdovirus* (formerly viral hemorrhagic septicemia virus, VHSV) remains the greatest problem. This virus was proven to infect wild and feral salmonids in European waters, most notably in direct connection with an active outbreak site in the vicinity [6], but VHSV was also identified in asymptomatic animals, such as European river lamprey (*Lampetra fluviatilis)* in Finland [7], or wild salmonids in Swiss rivers [8]. VHSV was also present in multiple wild saltwater species [9,10] and genotype IV of the virus caused massive multi-species mortality events in the North American Great Lakes in the early 2000s [11,12]. *Salmonid novirhabdovirus* (previously infectious hematopoietic necrosis virus, IHNV) is endemic in the Northwest Pacific off the coast of North America [13]. The presence of the virus in European wild fishes was mostly reported as asymptomatic, such as the findings in brown trout (*Salmo trutta fario*) in the Republic of Kosovo [14] and in Switzerland [8], or in pike (*Esox lucius*) in France [15].

Further pathogens seem to emerge as potential novel threats. Clinical infection caused by *Piscine orthoreovirus-3* (PRV-3) in rainbow trout was first described in Norway in 2013 [16], associated with anemia, heart and skeletal muscle inflammation and liver necrosis. Experimental infection with purified PRV-3 particles was recently proven to cause heart inflammation in rainbow trout [17]. The presence of the virus was confirmed in farmed rainbow trout in Germany [18], Italy and Denmark [19], in coho salmon (*Oncorhynchus kisutch*) in Chile [20] and in wild brown trout in Germany [21]. Kuehn et al. [21] argued that the virus is the main causative agent of the proliferative darkening syndrome (PDS), a disease affecting wild brown trout populations in pre-alpine rivers in Switzerland, Germany and Austria. PDS manifests itself with pathoanatomical signs such as skin darkening, hemorrhages and necrosis of liver, spleen and kidney resulting in massive species-specific die-offs of brown trout during the late summer months [22]. On the contrary, the presence of PRV-3 was described in clinically healthy brown trout [23], and brown trout showing PDS signs also tested PRV-3-negative in other cases [24]. Arndt et al. [24] connected the mortalities of brown trout in Austrian rivers with proliferative kidney disease (PKD), caused by the myxozoan *Tetracapsuloides bryosalmonae*. Additionally, Lewisch et al. [25] confirmed the presence of the parasite in wild fish from the majority of examined Austrian rivers, along with the clinical and pathoanatomical signs typical for PKD, such as darkened skin, abdominal distention and enlargement of the kidney and spleen [26]. 

With *T. bryosalmonae* recently confirmed as a pathogen of rainbow trout in one of the Czech recirculation aquaculture systems [27], and VHS and IHN outbreaks occasionally occurring in farmed animals in the country [4], we decided to evaluate the epizootiological situation of wild salmonids. The populations of these fish have been declining in the last decades and the presence of the selected pathogens has never been evaluated and remained unknown before the publication of this study. Furthermore, for the first time, we report the presence of the *piscine orthoreovirus-3* and *T. bryosalmonae* coinfection in the Czech Republic’s brown trout population and explore the potential role of wild salmonids as vectors of the aforementioned pathogens.

## 2. Results

### 2.1. Sampled Fish, Gross Pathology

In total, 266 salmonids were collected, mostly brown trout (n = 260) along with rainbow trout (n = 5) and brook trout (n = 1). The average weight of the fish was 67 g (SD ± 45.1 g) with the average total lenght of 173 mm (SD ± 51.9 mm). Most prevalent macroscopic lesions comprised swollen kidney (n = 36, Table 2) followed by enlarged spleen (n = 3), pale liver (n = 2) and saprolegniosis of adipose and caudal fins (n = 2). The presence of up to three leeches (*Piscicola* spp.) was noted on the fins of four fish from one location (Loučka, CZ-2082).

### 2.2. Serological Examination

In total, 110 sera were examined for the presence of specific antibodies against VHSV and IHNV using enzyme-linked immunosorbent assays (ELISA). In all samples, the pure absorbance value did not cross the threshold of 0.100 (Table 1). Therefore, all samples were declared negative for the presence of each of the two virus-specific antibodies. The titer of positive hyperimmune sera showed the values of 1:12,800 for VHSV and 1:6400 for IHNV and the average absorbance of a serum in 1:100 dilution analyzed on a negative antigen was determined as 0.150 (SD ± 0.034) for VSHV and 0.149 (SD ± 0.036) for IHNV.

### 2.3. Virological Examination

Virus isolation from 33 pooled samples containing material from a total of 266 fish showed no cytopathic effect on the fathead minnow (FHM) and epithelioma papulosum cyprini (EPC) cell lines after two rounds of a seven-day cultivation. The samples were therefore declared free of cultivable viral pathogens.

PCR assays targeting VHSV, IHNV, IPNV and SAV-2 showed negative results for each pooled sample tested. The conventional PCR for the detection of PRV-3 showed the presence of the virus in four of the analyzed samples (Table 1). Three samples from the location CZ-2019/1-3 were not tested for SAV-2 and PRV-3 due to material degradation during long-term storage.

**Figure 1:**
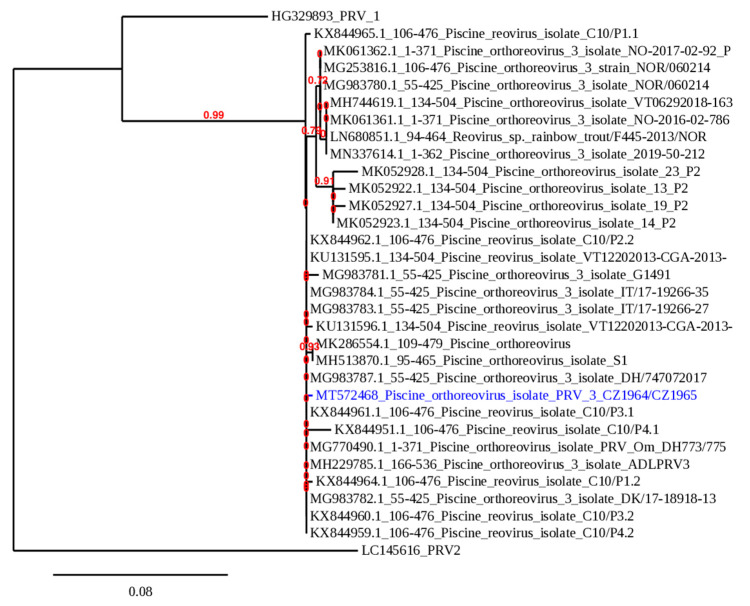
Phylogenetic analysis of PRV-3 isolates. A maximum likelihood phylogenetic analysis performed with PhyML on the 371 bp nucleotide sequence encoding a fragment of the segment S1 of the sigma 3 protein of the PRV-3 isolate from Czechia (GenBank ID: MT572468) which is marked in blue and other PRV-3 isolates available in GenBank. The branch length is proportional to the number of substitutions per site. The branch supporting values are indicated in red.

All samples were sequenced and the 371 bp sequences were analyzed phylogenetically with the corresponding PRV-3 sequences available in the GenBank database (Figure 1). 

All Czech isolates possessed the same sequence (GenBank ID: MT572468). Interestingly, the obtained sequence is a unique member of the PRV-3b subtype, with a very high level of identity (99.73%) to the sequences from several isolates from Germany, Italy, Denmark but also Chile. The real-time PCR assay for PRV-3 confirmed the presence of the virus in four respective samples and the number of copies of the target gene was calculated (Table 1). The normalized virus load was high, measuring from 1.27 × 10^3^ copies to 2.73 × 10^5^ copies.

### 2.4. Parasitological Examination

The results of the parasitological examination provided in Table 2 indicate that the parasite *T. bryosalmonae* was present in a varying percentage of fish at every location, even in the samples where the prevalence of gross renal lesions reached 0%.

## 3. Discussion

The original aim of this study was to analyze the possible presence of viral pathogens, especially the ones causing notifiable diseases, in Czech rivers. The assays for the detection of *piscine orthoreovirus-3* were added after multiple reports of the presence of the virus in European freshwater salmonids. Finally, the methods for the detection of *T. bryosalmonae* were included due to pathomorphological signs present in a large portion of the examined fish.

### 3.1. Viral Pathogens

The rivers selected for this study had various levels of connection to the farms rearing susceptible salmonids with a history of VHS or IHN outbreaks. None of the sites were tested during an active outbreak on any of the watersheds, due to practical reasons and to comply with the recommended water temperatures for VHSV/IHNV sampling events [28]. The delay between the last recorded outbreak and the first sampling was at least six months, which could have lowered the chances for the detection of the viruses. Even though VHSV and IHNV have historically been detected in European freshwater salmonids [8,14] and both viruses are causing occasional outbreak events in Czech fish farms [4], this study failed to prove the presence of the pathogens in the sampled brown trout specimens.

The lesser distribution of the viral pathogens in wild and feral fish compared with the fish reared in aquaculture establishments could have biological reasons, such as the lower susceptibility of brown trout to the viral infection [29], or environmental reasons, namely the lower population density and stress levels in wild fish compared with the farmed animals. Additionally, phylogenetic studies of the viral pathogens originating from various outbreak sites repeatedly showed that the transport of live fish and fish eggs is the main factor of the disease distribution amongst individual fish farms [3,30].

This study employed a range of sensitive methods for the detection of the virus capable of replication (cell culture), of viral nucleic acids (PCR) and of specific antibodies against VHSV and IHNV (ELISA). The negative results strongly suggested that the sampled animals were not infected with a notifiable disease-causing virus at the time of sampling and were not exposed to the viruses in concentrations causing a lasting antibody response [31,32]. However, the possibility of finding a viral pathogen at the examined locations still cannot be ruled out completely and could be improved, for example, via sampling of a larger number of fish or via repeated sampling in one location. Additionally, the pooling of the samples for the virological examination (deployed due to cost reasons), while still considering the manuals in European legislation for the surveillance of viral pathogens of fish [28], could have lowered the sensitivity of the assays.

As for the non-listed viral diseases, the presence of *salmonid alphavirus* has never been confirmed in Czech aquaculture, but its prevalence in European countries with rainbow trout production suggests the possibility of its spread via the trade of fish or eggs [33]. Mandatory monitoring of trout farms for the presence of infectious pancreatic necrosis virus was ongoing in the Czech Republic from 2008 to 2011, with zero cases reported positive [34]. The Czech National Reference Laboratory for Viral Diseases of Fish has registered only one isolate of the virus of Czech origin since 1997 (CAPM V-513), but the possibility of the presence of the virus in Czech rivers and aquaculture is still real and cannot be ruled out due to a lack of available data [8].

### 3.2. Piscine Orthoreovirus-3

None of the clinical and pathoanatomical signs corresponding to typical PRV-3 infection in rainbow trout, such as anemia, heart and skeletal muscle inflammation and liver necrosis [17] were observed in the fish examined in this study. However, the PRV-3 infection in sea trout (*Salmo trutta trutta*) mainly manifests as mild heart inflammation [35] which cannot be evaluated by gross pathology alone and would require a histopathological examination. This assessment should be added in future studies of PRV infections. Additionally, research on the direct influence of purified PRV-3 on the health status of brown trout would be of great importance, along with the potential role of this virus as a cofactor of brown trout disease. 

Since the role of PRV-3 as a pathogen of farmed rainbow trout has been established [17], it is necessary to evaluate the possible role of wild salmonids as vectors of the virus between potentially vulnerable rainbow trout farms. Infection experiments on Atlantic salmon and rainbow trout showed variations in PRV-3 sensitivity [36]; an infection trial comparing the susceptibility of rainbow trout and brown trout is therefore to be recommended. The rainbow and brook trout specimens collected in this study were probably escaped animals from a nearby aquaculture establishment because no population of these species was intentionally kept in the examined river (Moravian Fishing Association, personal communication), but the sample size (one and five specimens, respectively) was too small to draw any conclusions regarding the susceptibility of various salmonid species to this specific isolate of the virus.

Mortality events of a scale corresponding to the proliferative darkening syndrome description in the pre-alpine region have never been observed in the rivers represented in this study (Moravian Fishing Association, personal communication). Additionally, no clinical and pathoanatomical signs typical for PDS, such as darkening of the skin, anemia and liver necrosis, were observed in the examined fish, even in the rivers positive for PRV-3. It is, however, possible that the cases presented in this publication did not manifest clinical signs of infection due to the season, when the water temperature was not providing the best conditions for PDS development [21] or the environment of the Czech rivers was not suitable for PDS development. Further research of the possible relationship between PRV-3 and PDS is required [23,24]. 

### 3.3. Proliferative Kidney Disease

All pathoanatomical changes in moribund fish examined in this study corresponded to the typical proliferative kidney disease signs [26], but a possible role of coinfection cannot be ruled out completely because the PRV-3-positive fish were also positive for *T. bryosalmonae*. A potentiating effect of *T. bryosalmonae* and a viral agent (VHSV) on the changes of the immune system of salmonids was previously described by Gorgoglione et al. [37]. Other studies confirmed that a *Raphidascaris acus* infection prolonged PKD-related gross changes in brown trout kidneys [38].

The first cases of proliferative kidney disease in Czech aquaculture were reported during the 1980s [39], but no targeted surveillance of the distribution of this parasite in Czech wild salmonids has ever been conducted, despite the growing evidence of adverse effects of the infection on the brown trout populations in neighboring countries [24,25]. This study shows a widespread presence of the parasite in Czech rivers because at least some of the fish from all eight locations were tested positive for the presence of *T. bryosalmonae* in the kidney tissue during ten separate sampling events. This prevalence was confirmed by both diagnostic methods (IHC and qPCR) used, with the immunohistochemistry method showing significantly lower percentages of positive fish from each location compared with the results obtained by real-time PCR, which is a finding consistent with other published studies employing both methods of parasite detection [24,40]. This finding puts previous studies relying on histopathology or IHC for the confirmation of *T. bryosalmonae* infection in perspective and suggests that the percentages of positive samples, significant as they are, could be even higher when utilizing molecular techniques. 

Using histopathology and IHC, the PKD situation was best mapped in Switzerland, where *T. bryosalmonae* was found in 40–50% of the sampled rivers [41,42,43]. Studies on brown trout were also conducted in England (86% of rivers [44]) and Estonia (around 50% of rivers [45]). A recent study showed the presence of the parasite in 81% of the sampled rivers in Austria using conventional PCR [25]. In this study, the only two repeatedly sampled locations showed higher percentages of the examined fish displaying pathomorphological signs of PKD, along with greater numbers of samples tested positive for the presence of *T. bryosalmonae* using both IHC and qPCR in November, compared with the situation in April. Although the temperature in every sampling campaign presented was not optimal for the propagation of the parasite [43], the data confirmed the observed cycle of the infection in brown trout, with infection rates and pathoanatomical changes peaking in late summer [24], and a significant decline during cold temperature periods [25,27] when a partial or complete regeneration of renal tissue occurs [38]. To establish whether the reoccurring PKD symptoms in specific rivers are caused by reinfection of susceptible animals [38] or by a persistence of the parasite in clinically healthy fish [46,47] was beyond the scope of this study and would require additional repeated testing of selected locations. 

*T. bryosalmonae* acts as an immunomodulant [48] and can potentially cause up to 20% mortality in affected brown trout [26], with additional coinfections potentiating the pathologic effects [37,38]. Additionally, infections of European brown trout have a larger impact on the health of the infected fish, when compared with rainbow trout [40,49]. Despite the ubiquitous presence of the parasite in wild brown trout from Czech rivers, the local decline of the species cannot be blamed solely on this pathogen. However, additional factors contributing to the loss of fish [50], such as water pollution [51], river eutrophication [52] and a rising water temperature [53] also create suitable conditions for a better propagation of the pathogen.

To our knowledge, this study presents the first detection of *piscine orthoreovirus-3* in the Czech Republic and also represents the first described cases of proliferative kidney disease of wild brown trout from the Czech Republic.

## 4. Materials and Methods

### 4.1. Sample Collection and Preparation

Salmonid populations from eight Czech rivers with various connections to former VHS or IHN outbreak sites were sampled during ten separate sampling events in 2015–2017 in the season when the water temperatures did not exceed 14 °C, in accordance with European Legislation [28]. At each location, from 16 up to 32 specimens of brown trout and any additional salmonid species present were caught by electric fishing, with a total number of 266 fish (Table 3). The fish were measured and weighed on the spot and delivered alive to the laboratory.

The specimens were examined for the presence of clinical and pathoanatomical signs of disease, with an emphasis on changes of the kidneys. After blood samples were collected, the fish were stunned by a blow to the back of the head and killed by a spinal transection. During autopsy, spleen, heart and cranial kidney tissue samples were immediately homogenized for the virological examination and a caudal kidney sample from each fish was fixed for an immunohistochemical examination.

### 4.2. Serological Examination

Blood serum was collected from fish caught in the 5 rivers sampled in 2017. The serum of each individual fish was tested for the presence of specific antibodies against VHSV and IHNV by two separate enzyme-linked immunosorbent assays prepared in house according to Cinkova et al. [54]. The hyperimmune sera and concentrated antigens required for ELISAs were prepared from a recent Czech isolate of each virus and the isolates were also submitted to the Collection of Animal Pathogenic Microorganisms, Veterinary Research Institute, Brno, Czech Republic, under the numbers CAPM V-684 (VHSV) and CAPM V-629 (IHNV). The key reagents of the ELISAs were a conjugate of horseradish peroxidase with rabbit anti-trout antibodies prepared using the periodate method [55] and a chromogenic substrate containing 3.3′, 5.5′-tetramethylbenzidine (TMB2, TestLine Clinical Diagnostics, Brno, Czech Republic). Absorbance values were measured at a wavelength of 450 nm using the spectrophotometer SLT Spektra (Schoeller, Sevelen, Switzerland) and pure absorbance was calculated as the difference between the absorbance values of a positive and a negative antigen.

### 4.3. Virological Examination

Spleen, heart and cranial kidney tissues from up to 12 animals were pooled for a total of 33 pooled samples. The tissues were mechanically lysed, suspended in a ten-fold volume of Minimal Essential Medium with Eagles salts MEM HEPES Modification (Sigma-Aldrich, St. Louis, MO, USA) cell culture medium and centrifuged at 3000 G for 14 min at 4 °C. The supernatant from homogenized pooled tissue samples was incubated overnight at 4 °C in a 10:1 ratio with Penicillin-Streptomycin Solution 100X (Biosera, Boussens, France) and then incubated in a 24-well open system on two different cell lines—fathead minnow (FHM) and epithelioma papulosum cyprini (EPC) (Sigma-Aldrich, St. Louis, MO, USA) at 15 °C for 7 days using the Minimal Essential Medium with Eagles salts MEM HEPES Modification medium with 10% addition of fetal bovine serum (Biochrom, Berlin, Germany) and l-Glutamine 200 mM (Lonza, Basel, Switzerland). The inoculated cell line was 24 h old at the moment of infection and the presence of an intact monolayer was confirmed in each well before the experiment. Material from the first passage was incubated under the same conditions for an additional 7 days. 

All samples were examined for the presence of viral pathogens of salmonids using conventional and real-time PCR. The list of specific assays used for each pathogen is shown in Table 4. For nucleic acid extraction, the QIAamp Viral RNA Mini Kit (Qiagen, Hilden, Germany) was used. All samples positive in conventional PCR were prepared for Sanger sequencing performed by LGC Genomics (Berlin, Germany). The obtained nucleotide sequences of the PRV-3 segment S1 were aligned with piscine orthoreovirus sequences available in GenBank using tools available at www.phylogeny.fr [56]. Sequences were aligned with MUSCLE and curated with Gblocks. A maximum likelihood phylogenetic analysis was performed with PhyML and the phylogenetic tree was rendered with TreeDyn. One PRV-1 isolate from Norway (GenBank ID: HG329893) was used for comparison, and the tree was rooted using a PRV-2 sequence (GenBank ID: LC145616) as an outgroup (Figure 1). The approximate viral load was measured in all positive samples by quantitative PCR. The quantification was performed using the plasmid-based standard curve method with normalization of the target RNA copies with 10,000 copies of trout elongation factor 1α [57].

### 4.4. Parasitological Examination

The presence of kidney swelling was evaluated as either 0 (not present) or 1 (present). The caudal kidney tissue sample of each fish was fixed in 10% neutral buffered formalin for the immunohistochemical examination, and in the samples CZ_2072–CZ_2092, additional kidney tissue was fixed in 70% ethanol for DNA isolation. Samples of the formalin-fixed caudal kidney were examined by immunohistochemistry using the monoclonal anti-*Tetracapsuloides bryosalmonae* antibody (AquaMAb-P01, Aquatic Diagnostics, Scotland, UK; [63]), with each kidney sample declared as PKD-positive or -negative. For all kidney samples, ten microscopic fields (200× magnification) per slide were randomly selected, the number of parasites per field was manually counted and the mean number of parasites for all ten fields was calculated [64]. Kidney tissues fixed in 70% ethanol (samples CZ_2072–CZ_2092) or the formalin-fixed paraffin wax-embedded kidney tissues (samples CZ_1964–CZ_1987) were used for molecular detection of the presence of *T. bryosalmonae* nucleic acid using the real-time PCR assay by Bettge et al. [62]. DNA for these analyses was extracted using the DNeasy Blood and Tissue kit (QIAGEN, Hilden, Germany), following the manufacturer’s instructions.

## Figures and Tables

**Table 1:** Results of the virological analysis of the samples.

Sample River	EPC Cells	FHM Cells	VHSV PCR	VHSV ELISA	IHNV PCR	IHNV ELISA	IPNV PCR	SAV-2 PCR	PRV-3 PCR	PRV-3 Conc. *
CZ_1964_1 Oslava	Neg.	Neg.	Neg.	-	Neg.	-	Neg.	Neg.	**Pos.**	2.73 × 10^5^
CZ_1964_2 Oslava	Neg.	Neg.	Neg.	-	Neg.	-	Neg.	Neg.	**Pos.**	2.59 × 10^4^
CZ_1964_3 Oslava	Neg.	Neg.	Neg.	-	Neg.	-	Neg.	Neg.	**Pos.**	6.66 × 10^3^
CZ_1965_1 Jihlava	Neg.	Neg.	Neg.	-	Neg.	-	Neg.	Neg.	Neg.	-
CZ_1965_2 Jihlava	Neg.	Neg.	Neg.	-	Neg.	-	Neg.	Neg.	Neg.	-
CZ_1965_3 Jihlava	Neg.	Neg.	Neg.	-	Neg.	-	Neg.	Neg.	**Pos.**	1.27 × 10^3^
CZ_1965_4 Jihlava	Neg.	Neg.	Neg.	-	Neg.	-	Neg.	Neg.	Neg.	-
CZ_1965_5 Jihlava	Neg.	Neg.	Neg.	-	Neg.	-	Neg.	Neg.	Neg.	-
CZ-1986/1-4 Svratka	Neg.	Neg.	Neg.	-	Neg.	-	Neg.	Neg.	Neg.	-
CZ-1987/1-3 Loučka	Neg.	Neg.	Neg.	-	Neg.	-	Neg.	Neg.	Neg.	-
CZ-2019/1-3 Losenický	Neg.	Neg.	Neg.	-	Neg.	-	Neg.	-	-	-
CZ-2072/1-2 Sázava	Neg.	Neg.	Neg.	<0.1	Neg.	<0.1	Neg.	Neg.	Neg.	-
CZ-2079/1-2 Svratka	Neg.	Neg.	Neg.	<0.1	Neg.	<0.1	Neg.	Neg.	Neg.	-
CZ-2079/3-4 Loučka	Neg.	Neg.	Neg.	<0.1	Neg.	<0.1	Neg.	Neg.	Neg.	-
CZ-2082/1-3 Svitava	Neg.	Neg.	Neg.	<0.1	Neg.	<0.1	Neg.	Neg.	Neg.	-
CZ-2090/1-3 Dyje	Neg.	Neg.	Neg.	<0.1	Neg.	<0.1	Neg.	Neg.	Neg.	-

* Quantification via plasmid-based standard curve with normalization of the target RNA copies with 10,000 copies of trout elongation factor 1α; EPC: cultivation on the epithelioma papulosum cyprini cell line, FHM: cultivation on the fathead minnow cell line.

**Table 2:** Results of the parasitological examination focused on *Tetracapsuloides bryosalmonae*.

Sample River	Fish (n)	Swollen Kidney (%)	IHC Positive Fish (%) Parasites (n) * (Mean ± SD)	qPCR Positive Fish (%) ** Mean Ct ± SD
CZ-1964 Oslava	32	3.1	15.6 0.68 ± 0.70	96.9 *** 28.08 ± 2.77
CZ-1965 Jihlava	28	7.1	14.3 3.45 ± 3.14	100.0 *** 24.50 ± 1.63
CZ-1986 Svratka	32	0	9.4 0.50 ± 0.32	25.0 *** 31.73 ± 1.13
CZ-1987 Loučka	30	0	10.0 0.80 ± 1.04	73.3 *** 27.07 ± 3.14
CZ-2019 Losenický	32	43.8	62.5 24.02 ± 22.63	6.3 *** 31.0 ± 0,84
CZ-2072 Sázava	17	17.6	35.3 8.60 ± 8.88	94.1 **** 23.98 ± 3.26
CZ-2079/1,2 Svratka	16	18.8	50.0 3.39 ± 3.39	81.3 **** 26.77 ± 2.81
CZ-2079/3,4 Loučka	17	17.6	64.7 24.54 ± 59.75	76.5 **** 28.54 ± 3.28
CZ-2082 Svitava	30	0	6.7 1.40 ± 1.84	56.7 **** 29.85 ± 2.39
CZ-2090 Dyje	32	21.9	21.9 3.96 ± 6.13	59.4 **** 27.91 ± 2.57

* The average number of parasites in the field of view, magnification 200×, 10 fields per slide, ** detection limit: Ct 32 (inclusive), *** DNA extracted from formalin-fixed, paraffin wax-embedded kidney tissue, **** DNA extracted from 70% ethanol-fixed kidney tissue; IHC: immunohistochemistry, SD: standard deviation.

**Table 3:** Sites, dates and animals sampled for this study.

Sample	River	Date	GPS	*ST* (n)	Other (n)	Temp. (°C)	Sera (n)
CZ-1964/1-5	Oslava	27 October 2015	49°21′59.0″ N 16°01′06.1″ E	26	*OM* 1, *SF* 5	4.0	-
CZ-1965/1-3	Jihlava	28 October 2015	49°05′52.8″ N 16°13′18.2″ E	28	-	11.0	-
CZ-1986/1-4	Svratka	18 April 2016	49°28′59.5″ N 16°20′22.7″ E	32	-	6.8	-
CZ-1987/1-3	Loučka	18 April 2016	49°23′25.0″ N 16°18′08.2″ E	30	-	9.9	-
CZ-2019/1-3	Losenický	26 October 2016	49°34′06.9″ N 15°46′27.0″ E	32	-	9.0	-
CZ-2072/1-2	Sázava	25 September 2017	49°33′16.2″ N 15°50′55.4″ E	17	-	12.8	15
CZ-2079/1-2	Svratka	2 October 2017	49°28′59.5″ N 16°20′22.7″ E	16	-	7.7	16
CZ-2079/3-4	Loučka	2 October 2017	49°23′25.0″ N 16°18′08.2″ E	17	-	7.1	17
CZ-2082/1-3	Svitava	8 November 2017	49.2959953 N, 16.6644897 E	30	-	7.5	30
CZ-2090/1-3	Dyje	8 November 2017	48.8523892 N, 15.8646003 E	32	-	3.3	32

ST: *Salmo trutta fario*, OM: *Oncorhynchus mykiss*, SF: *Salvelinus fontinalis*.

**Table 4:** PCR assays used in this study.

Target Organism	Method	Author
VHSV	Real-time RT-PCR	Jonstrup et al., 2013 [58]
IHNV	Real-time RT-PCR	Purcell et al., 2013 [59]
IPNV	Conventional RT-PCR	Orpetveit et al., 2010 [60]
SAV-2	Conventional RT-PCR	Hodneland and Endresen, 2006 [61]
PRV-3	Real-time RT-PCR	Adamek et al., 2019 [18]
PRV-3	Conventional RT-PCR	Olsen et al., 2015 [16]
*T. bryosalmonae*	Real-time PCR	Bettge et al., 2009 [62]
*Salmo trutta-ef1α*	Real-time PCR	Dietrich et al., 2015 [57]
